# Chest Wall Reconstruction with Porcine Acellular Dermal Matrix (Strattice) and Autologous Tissue Transfer for High Risk Patients with Chest Wall Tumors

**DOI:** 10.1097/GOX.0000000000001703

**Published:** 2018-05-18

**Authors:** Haitham H. Khalil, Maninder Kalkat, Marco N. Malahias, Saif Rhobaye, Tarek Ashour, Madava G. Djearaman, Babu Naidu

**Affiliations:** From the * Department of Oncoplasty and Reconstructive Surgery, Good Hope Hospital, Heart of England NHS Foundation Trust, Birmingham, West Midlands, United Kingdom;; † Department of Thoracic Surgery, Heartlands Hospital, Heart of England NHS Foundation Trust, Birmingham, West Midlands, United Kingdom;; ‡ Department of Radiology, Heartlands Hospital, Heart of England NHS Foundation Trust, Bordesley Green East, West Midlands, United Kingdom.

## Abstract

**Background:**

Tenets of chest wall reconstruction for malignancy are well known; however, the insertion of synthetic prosthetic material to achieve rigid and nonrigid skeletal reconstruction could be associated with more complications including infection and extrusion especially in high risk patients. This includes fungating, infected tumors, previous radiotherapy, scars, smoking, diabetes, and morbid obesity. Bioprosthesis Acelluar Dermal Matrix Strattice would be a substitute to resist infection and provide stable coverage.

**Methods:**

A retrospective data analysis study on 8 high-risk patients who underwent chest wall resection and reconstruction with bioprosthesis (Strattice) with/without titanium plates in the period between 2012 and 2017 was performed. Patient’s demographics, risk factors, clinico-pathological, radiological, operative details, adjuvant therapy, postoperative morbidity, and follow-up data were recorded.

**Results:**

Sarcoma was the pathology in 7 and recurrent breast cancer in 1 with age range from 21 to 71 years (mean, 50) and preponderance of female patients (n = 5). Defects were located anterior/anterolateral with size ranging from 270 to 1,050 cm^2^ (mean, 511). Reconstruction was performed using Strattice only in 4 patients, whereas in 4 it was combined with titanium plates. All patients required flap reconstruction (3 pedicled and 5 free). The follow-up ranged from 9 to 52 months (mean, 24.8). Minor complications occurred in 2 patients; however, good functional outcome was achieved in all.

**Conclusion:**

Strattice would act as a safe alternative modality for chest wall reconstruction to resist infection in high-risk patients with extensive defects. It should be considered as a valuable tool in the armamentarium of chest wall reconstruction.

## Abstract 2

Supplemental Digital Content is available in the text.

## INTRODUCTION

Surgery is considered the corner stone for management of chest wall malignancy.^1–3^ The wide resection resulting in large chest wall defects can lead to instability, interference with respiration with the potential increase in postoperative morbidity and mortality.^4,5^ The principles of chest wall reconstruction are well known, radical resection ensuring adequate tumor-free margins, maintenance of chest wall stability, lung functional, and acceptable cosmetic results.^1,2^ However, the insertion of prosthetic material to achieve rigid and nonrigid skeletal reconstruction could be associated with more complications including surgical-site infection (SSI), wound dehiscence, and possible extrusion of prosthesis especially in high-risk patients.^3,6,7^ Uni/multivariate analysis of predictors of complications has been previously discussed in the literature including general factors (diabetes, immune-suppression, morbid obesity, smoking) and local factors (previous radiotherapy and surgery, fungating and infected tumors).^1,6,8^ Various materials have been reported to be used for either rigid or nonrigid chest wall reconstruction to achieve functional chest wall stability including mainly synthetic meshes [Polyprolene (Marlex) poly tetrafluoroethylene (Gore-Tex)], Methylmethacrylate sandwich mesh, Stratos osteosynthesis system comprising titanium bars and clips and other metallic substitutes.^3,9–11^ On the other hand, there is sparse evidence reported in the literature mainly reported cases on the use of biological mesh (Permocal, Alloderm, and Strattice) both in clinical practice and animal model.^7,12–17^ We report our case series experience using acelluar dermal matrix (ADM) Strattice in patients at increased risks of synthetic prosthetic mesh–related complications due to mainly local factors including infected fungating and necrotic tumors, previous surgery and perioperative radiotherapy/chemotherapy and to a lesser extent general factors as smoking, morbid obesity, and diabetes.

## MATERIALS AND METHODS

Prospectively collected data from 8 consecutive cancer patients who underwent chest wall resection and reconstruction with Strattice (ADM) biological mesh in the period between January 2012 and April 2017 were reviewed. The indication to consider bioprosthetic material Strattice included high-risk patients having contamination of the operative field (fungating, infected, and necrotic tumors), previous local radiotherapy and surgery and/or associated general risk factors as diabetes, active smoking, chemotherapy, and morbid obesity. All patients were managed through a multidisciplinary approach including thoracic surgeon (M.K.) and reconstructive surgeon (H.K.) to optimize their perioperative treatment plan. This included preoperative preparation, pulmonary function test to assess fitness, radiological investigations including computed tomographic (CT) scan and/or angiography and magnetic resonance imaging to plan the surgical resection, possible skeletal and soft-tissue reconstructive options and oncological adjuvant therapy.

### Data Collection

A prospectively maintained database was used to identify the patients, and their records were assessed retrospectively. The demographic information and potential identifiable risks and comorbidities, previous history of malignancy, and treatment were collected. Tumor characteristics and details of adjuvant therapy including either chemotherapy or radiotherapy were also documented. The results of the radiological diagnostic workup included standard chest x-ray, CT scan, and magnetic resonance imaging to delineate the extent of the tumor in the chest wall, and involvement of adjacent surrounding structures was noted. The operative details identified the numbers of ribs and costochondral junctions along with other structures that were resected as part of the tumor extipiration and included intercostal muscles, chest wall muscles, lungs, diaphragm, and overlying soft tissue. The sizes of the skeletal and soft-tissue defect were recorded in all patients. Documentation of whether Strattice was used solely or in adjunct with titanium plates and rib clips (Strasbourg Thoracic Osteosyntheses System; MedXpert Gmbh, Heitersheim, Germany) to restore chest wall integrity was noted. In addition, the methods of soft-tissue coverage were also documented. The postoperative outcomes in terms of length of stay, complications particularly SSI, wound dehiscence, chest wall stability, and respiratory function were identified from the patient records.

### Operative Consideration

All patients included were considered for surgery after been reviewed by the multidisciplinary thoracic oncoplastic team in the sarcoma and breast multidisciplinary team meeting (MDT). The resection and reconstruction were performed as a 1-stage procedure in all patients with a 2 team approach. In the majority of cases (5/8), the 2 teams worked simultaneously when free flap reconstruction was performed, whereas in 2 (2 of 8), the thoracic surgeons initially performed the resection and restored the skeletal wall integrity; this was followed by harvesting and insetting of the selected regional flap. On the other hand, in 1 patient, the reconstructive team harvested the selected regional flap first to facilitate tumor extipiration and also salvage the regional flap from being damaged through the conventional thoracic approach. Post tumor resection chest wall stability was achieved using firm Strattice ADM (30 × 30 or 15 × 15 cm), which was washed in vitro with 5 L of saline to remove any chemical preservatives, followed by fashioning of the sheet to the defect (onlay technique) and securing it to the surrounding rib edges with interrupted Ethibond 2/0 sutures. Titanium plates and rib clips (Stratos osteosynthesis MedExpert) were used in addition to the Strattice ADM in 4 patients due to the extent of the resection and the location of the tumor being anterolateral to maintain structural support and stability. The site, size of the defect, availability of tissues, and preference of the reconstructive surgeon determined the choice of the soft-tissue flap. Soft-tissue reconstruction was achieved utilizing loco-regional pedicled muscle flaps as latissimus dorsi with or without split-thickness graft. Free microvascular tissue was indicated in more extensive resection with large surface area or when loco-regional flaps deemed not feasible due to direct tumor involvement, vascular compromise, and previous radiotherapy or surgery. We were mindful of creating a suitable window in the Strattice sheet in the cases where the internal mammary pedicle was used as the recipient pedicle for the free flap to avoid any compromise of the vascular supply postmicrovascular anastomosis. As a routine, all surgical sites were copiously irrigated with pulse-jet lavage using 3 L of saline (0.9% Nacl) solution to mechanically remove any debris or loose diathermized tissue and to maintain moisture of the Strattice. A chest wall tube was placed in the thoracic cavity, whereas suction drains were inserted deep into the flap and subcutaneous tissue. The duration of the drainage depended on the clinical, chest x-ray, and biological follow-up. Intraoperative collagen implant impregnated with aminoglycoside antibiotic gentamicin (Collatamp, EUSA Pharma, Hempstead, UK) was inserted in the operative field before closure. All 8 patients were transferred to intensive therapy unit with 6 patients (6 of 8) extubated at the end of surgery and 2 within 12 hours. Standard flap observation chart protocol was followed, including Doppler signal, skin color, temperature, and capillary refill. At induction, all patients received antibiotics intravenous therapy, determined by appropriate tissue penetrability with special reference to bone and joint infections for prosthetic device^18^ and continued routinely for 48 hours postoperatively followed by oral therapy for the duration of time the drains remained in situ. All patients received structured physiotherapy and rehabilitation program for both chest and donor site depending on flap selection.

## RESULTS

**Fig. 1.**
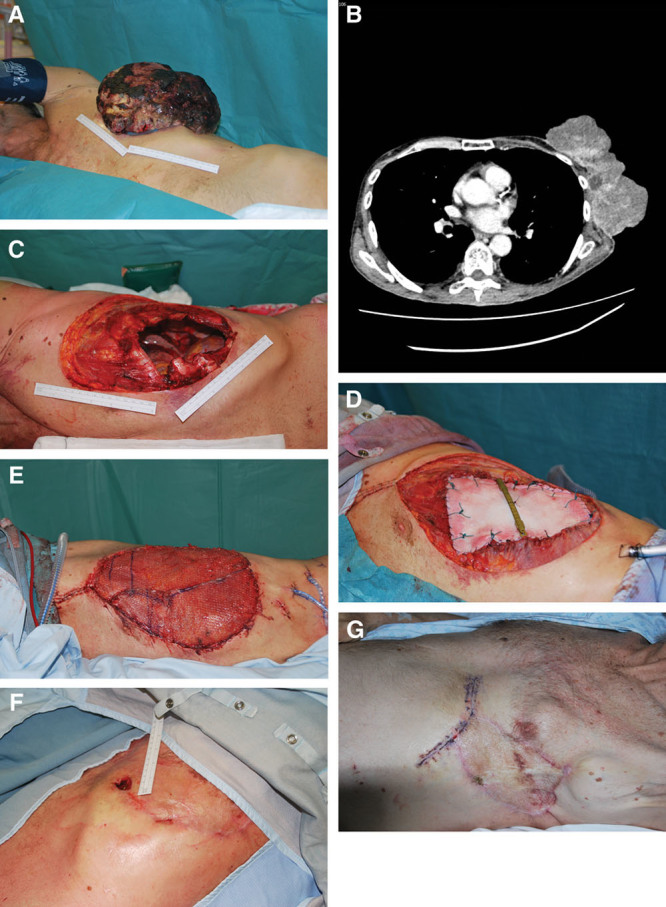
A, Large undifferentiated fungating necrotic soft-tissue sarcoma left chest wall in a 53-year-old smoker male patient. B, CT scan defining the extent of the lesion with involvement of underlying ribs and intercostals space necessitating resection of ribs to achieve negative surgical margins; C, Intraoperative photograph post en bloc tumor resection including 4 ribs demonstrating the skeletal defect exposing underlying thoracic viscera. D, Intraoperative photograph showing reconstruction of the skeletal defect using Strattice ADM used with STRATOS bar. E, Intraoperative photograph showing soft-tissue coverage achieved with lattissmus dorsi muscle flap and meshed skin graft. F, 10 Months postoperative photograph showing 1 × 2 cm distal wound dehiscence postradiotherapy. G, Postoperative photograph 3 weeks postdebridement and secondary closure of the dehiscence area. Note the full maturation of the graft.

**Fig. 2.**
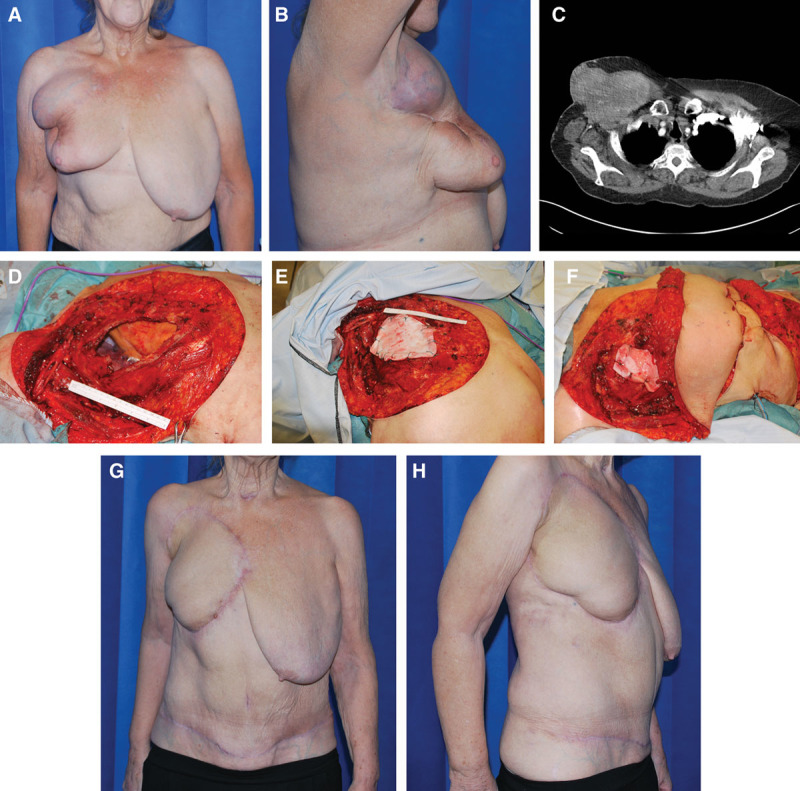
A and B, Preoperative photograph showing radiation induced sarcoma of right upper chest wall in a 71-year-old female patient post breast cancer treatment. High risk factors included smoking, diabetes, previous radiotherapy, and previous chronic deep abscess (front-lateral view). C, CT scan defining the extent of the lesion with involvement of underlying ribs and intercostals space necessitating resection of ribs to achieve negative surgical margins. D, Intraoperative photograph post en bloc tumor resection including 3 ribs demonstrating the extent of skeletal and soft-tissue resection exposing underlying thoracic viscera, axillary neurovascular bundle and brachial plexus. E, Intraoperative photograph showing reconstruction of the skeletal defect using nonrigid reconstruction with Strattice ADM only. F, Intraoperative photograph postmicrovascular anastomosis of free MSTRAM type I using the internal mammary vascular pedicle as recipient vessels to provide soft-tissue coverage of the chest wall defect. G and H, 12 Months postoperative photograph showing complete survival of the flap with primary healing and restoration of chest wall anatomy (front-lateral view).

The patient, tumor, and chest wall defect characteristics including surgical management and outcomes for the cases are summarized in Table 1. The risks for SSI included fungating, infected and necrotic tumors, previous local radiotherapy, and surgical scar along with general risk factors as diabetes, active smoking, chemotherapy, and morbid obesity. The tumor tissue was recurrent breast cancer in 1 and sarcoma in 7 and with age range from 21 to 71 years (mean, 50) and preponderance of female patients (n = 5). Resultant defects were located anterior, lateral, and anterolateral with sizes ranging from 270 to 1,050 cm^2^ (mean, 511).The average number of ribs resected with adjoining costochondral cartilage was between 3 and 10 (median, 4). In addition, partial sternectomy was performed in 1 patient and complete sternal resection in another one. Adequate clear surgical resection margins were achieved in all the patients. The resultant chest wall defect was repaired using Strattice in all patients. In 4 patients, the defects were large, crossing midline and required additional rigid reconstruction using titanium plates and rib clips (Stratos). The repaired defects were covered in all patients with soft-tissue flaps successfully achieved with free microvascular tissue transfer in 5 patients [muscle sparing transverse abdominus myocutaneous (MSTRAM) type I (n = 4) and tensor fasciae latae (TFL; n = 1)], whereas in 3 pedicled latissimus dorsi muscle with split-thickness graft and musculocutaneous flap were performed. The recipient vessels for the microvascular anastomosis were internal mammary pedicle (n = 4) and thoracodorsal pedicle (n = 1; Figs 1–5; **see video, Supplemental Digital Content 1**, demonstrating the multidisciplinary 2-team simultaneous approach for enbloc resection of a fungating infected large chondrosarcoma of the anterior chest wall. This video is available in the “Related Videos” section of PRSGlobalOpen.com or at http://links.lww.com/PRSGO/A722).

**Table 1.**
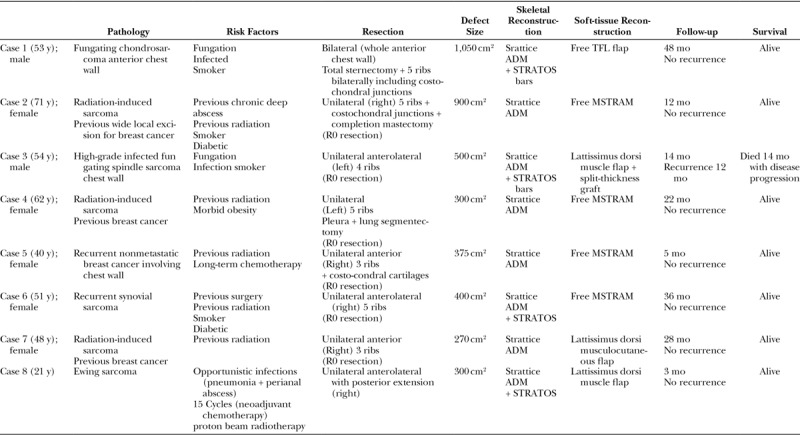
The Patient, Tumor, and Chest Wall Defect Characteristics Including Surgical Management and Outcomes

**Video Graphic 1.**
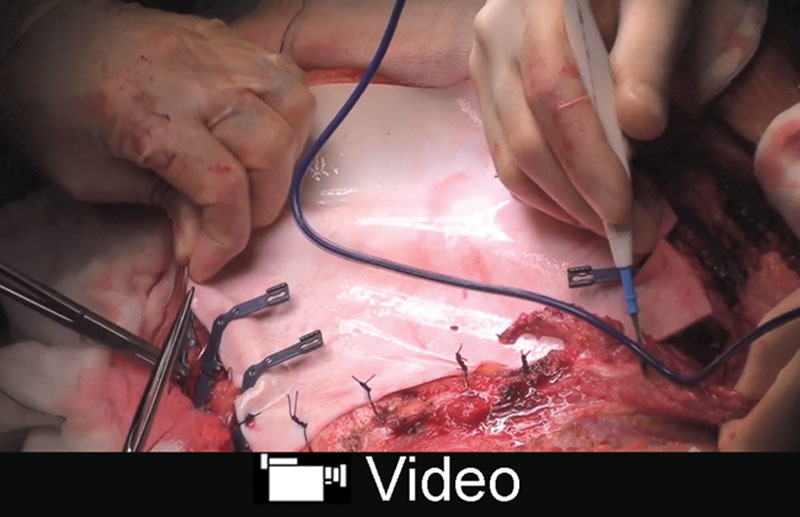
See video, Supplement Digital Content 1 demonstrating the multidisciplinary 2-team simultaneous approach for enbloc resection of a fungating infected large chondrosarcoma of the anterior chest wall. Strattice ADM and titanium plates (Stratos) were used to restore the skeletal chest wall integrity while soft-tissue reconstruction was achieved using free TFL flap. The resection included the sternum with the adjacent 7 costochondral cartilages and ribs on each side with exposure of the underlying thoracic viscera. Restoration of the chest wall integrity was subsequently achieved with Strattice ADM and titanium plates (Stratos). The free TFL flap provided soft coverage using the internal mammary vascular pedicle as the recipient vessels. The postoperative photographs demonstrate the complete survival of the flap primary wound healing and restoration of the chest wall integrity, whereas the postoperative CT scan reveals the maturation of the Strattice ADM providing stable chest wall reconstruction. This video is available in the “Related Videos” section of PRSGlobalOpen.com or at http://links.lww.com/PRSGO/A722.

Two patients experienced minor complications, 1 developed wound breakdown and superficial SSI 10 months postoperatively after receiving radiotherapy. This required minor surgical debridement with secondary closure. The same patient developed local recurrence and distant metastases (month 12 postoperatively) and was referred to the palliative team. The other patient developed superficial minor distal flap necrosis, which required debridement and flap readvancement day 14 postoperatively; in neither patients, the Strattice was exposed. During the follow-up period of 9–52 months (mean, 24.8), there was no local recurrence or distant metastases in any of the other patients with clinically good chest wall function. No readmissions were encountered within the 30 days from discharge. One patient died at 14 months due to systemic disease progression.

## DISCUSSION

The synthetic prosthesis mesh is used in the majority of chest wall reconstruction following resection of tumors. However, there is an increased risk of SSI, particularly if used in compromised surgical fields including fungating infected necrotic tumors, previously local radiotherapy and surgical scarring and in patients with general risk factors as diabetes, active smoking, chemotherapy, and morbid obesity.^7^ Furthermore, if infection and/or exposure of mesh ensue in the event of being exposed due to dehiscence or breakdown of overlying skin, surgical removal is often required. Observation from previously published reports demonstrated the relatively high infection rate (6–22%) in chest wall reconstruction in noncontaminated defects with up to 42% requirement of removal of synthetic mesh.^6^ In this series, of extensive chest wall defects (mean, 511 cm^2^) in compromised surgical fields, Strattice ADM was used to substitute synthetic mesh to resist infection. The general consensus accepted for chest wall skeletal reconstruction that we also applied in our series is that any defect less than 5 cm in size in any location, and those up to 10 cm in size posteriorly do not need rigid reconstruction for functional reasons. On the other hand, posterior defects in proximity to the tip of the scapula and larger lesions mostly anterior and lateral are likely to produce paradoxical chest wall motion therefore requires rigid reconstruction.^1,6,10,11^ Four patients in our series required additional structural support with rigid skeletal reconstruction using titanium plates (Stratos) due to the extent and location of the defects in the anterolateral chest wall region to maintain chest wall integrity. To date, there are few case reports demonstrating the use of Strattice in chest wall reconstruction.^14,16,17^ Alternatively, other bioprosthetic meshes as Alloderm and Permacol in both human and animal models have shown to be efficacious in similar scenarios.^7,12,13^ The advantages of biological mesh materials over synthetic material have been reported, which includes superior handling properties, reduced visceral adhesions, resistance to infection, better integration into the autologous tissue due to host cell infiltration, and revascularization while maintaining structural integrity.^7,13^ In addition, bioprosthesis has better tolerance to exposure due to tissue breakdown, hence can be managed conservatively rather than surgical extipiration.^7,13,19^ Despite the ability of both Alloderm and Strattice to become revascularized and resist infection, previous case reports have highlighted the superiority of porcine-derived over human-derived biological mesh including less elasticity to avoid stretch, been harvested in larger and consistent sheets achieving coverage of larger defects with a single sheet.^15,17,20^ This was in concordance with our results, as 1 sheet was used per patient notwithstanding that the defect sizes reconstructed in this series were relatively larger from previously reported cases. The relatively longer follow-up (mean, 24.8 months) when compared with previous reports illustrated the durability of the functional properties of a remodeled mature biologic scaffold in terms of strength and stretch. During the follow-up period, all patients showed satisfactory functional outcome assessed clinically and with pulmonary function testes complimented with equally cosmetic satisfactory cosmetic outcomes. Hedgire et al.^21^ reported on significant enhancement of Alloderm Biologic Mesh Spacer in the abdomen and pelvis detected on follow-up multi detector CT scan through progressive rise in Hounsfield unit values. This has been attributed to the possible revascularization of the ADM as supported by other reports.^22^ In our study, this was also observed in the CT scan follow-up performed postoperatively in 4 patients, which revealed well-enhanced scar tissue denoting revascularization and maturation providing stable coverage. Arguably other synthetic mesh materials would have been used; however, there would be an increased risk of infection of these synthetic meshes within this compromised surgical field and their intolerance for contamination and /or cutaneous exposure, which has been highlighted in other studies.^7,8,14^ Eventually, this would lead to detrimental consequences as a result of the requirement of mesh removal, which has been also highlighted in previous studies.^6,7,14^ In his case report, Brunbjerg et al.^14^ reported the use of Strattice successfully in chest wall reconstruction in a patient with multiple recurrent breast cancer, chemotherapy, and radiotherapy requiring multiple chest wall resection and reconstruction with previous failure and extrusion of the polypropylene mesh. We previously reported the value of multidisciplinary thoracic oncoplastic approach in the reduction of infection (2.8%) in the presence of synthetic prosthetic material in noncontaminated chest wall defects.^3^ Adopting the same approach in this relatively small cohort of high-risk patients, the pertinence of bioprosthetic mesh is seen to be associated with decreased incidence of SSI, less extrusion rate without any compromise of the chest wall stability that has also been supported by other studies.^6,7,14^ Observation from previously published reports demonstrated the importance of the proximate collaboration between several disciplines in managing these patients; however, it underscores the occurrence of the wound morbidity even in the presence of soft-tissue coverage for rigid reconstruction ranging from 9% to 25%,^1,8^ whereas in other series, this relationship was not highlighted.^9,23^ The recruitment of well-vascularized soft tissue is paramount to provide coverage of the prosthesis, prevent infection, seal the pleural space, protect underlying viscera, obliterate dead space, contribute to structure integrity and healing, and enhance aesthetic outcome.^1–3,7,24,25^ A wide variety of flaps could be used including loco-regional or free flaps depending on the site, size, and availability of tissues from donor sites and the available expertise.^3,7,26^ Soft-tissue coverage was performed in all patients in this series due to the extent of the cutaneous defects and to provide protection for the underlying Strattice and titanium plates. The majority of the patients required free flap including MSTRAM and TFL (5 of 8; 62.5%) when loco-regional flaps deemed not feasible, extent of the resection and due to the logistics of positioning the patients to allow thoracic and reconstructive surgeons working simultaneously. The efficacy of the xenogeneic nature of biological mesh has been questionable in previous reports^7,13,27,28^; however, with the rapidly increasing use of porcine-derived ADM in several indications including breast, abdominal, head and neck, dural reconstruction and static reconstruction postfacial nerve paralysis over the last decade has demonstrated its advantages.^8,29–33^

The current literature is largely limited to case studies, and to the authors best knowledge, this would be the largest series from a single institute to be reported with long-term follow-up addressing the decreased incidence of SSI and durability of chest wall reconstruction with the use of Strattice ADM in high risk patients. However, the limitation for this study is the relatively low number of patients from a single institute mainly due to the rarity of these presentations, the lack of available control group for direct comparison, and the requirement of potential objective analysis comparing pre- and postsurgery chest wall biomechanics. This study is driven to evaluate the indications and functional outcomes in patients undergoing chest wall reconstruction with bioprosthesis Strattice ADM, which would act as a safe alternative modality for chest wall reconstruction to resist infection in high risk patients with extensive defects. It should be considered as a valuable tool in the armamentarium of chest wall reconstruction.

## ACKNOWLEDGMENTS

The authors acknowledge the efforts and expert input of the Medical Illustration Team at Heart of England NHS Foundation Trust.

## Supplementary Material
